# Dynamic quantitative nonenhanced magnetic resonance angiography of the abdominal aorta and lower extremities using cine fast interrupted steady-state in combination with arterial spin labeling: a feasibility study

**DOI:** 10.1186/s12968-019-0562-3

**Published:** 2019-09-02

**Authors:** Emily A. Aherne, Ioannis Koktzoglou, Benjamin B. Lind, Robert R. Edelman

**Affiliations:** 1 Department of Radiology, NorthShore University HealthSystem, Walgreen Building, G507 2650 Ridge Ave, Evanston, USA; 2 McGaw Medical Center of Northwestern University, 2650 Ridge Ave Evanston, Chicago, IL 60201 USA; 3 University of Chicago Pritzker School of Medicine, Chicago, USA; 4 Department of Surgery, NorthShore University HealthSystem, 9650 Gross Point Rd Ste 4900, Skokie, Evanston, IL 60076 USA; 5 Northwestern University Feinberg School of Medicine, Chicago, USA

**Keywords:** Peripheral artery disease, Magnetic resonance imaging, Flow measurement, Angiography, Fast interrupted steady-state, Quiescent interval slice-selective

## Abstract

**Background:**

Cine fast interrupted steady-state in combination with arterial spin labeling is a recently described nonenhanced magnetic resonance angiography (MRA) technique that relies on bolus tracking for time-resolved digital subtraction angiography-like displays of blood flow patterns. We evaluated the feasibility of applying this technique to display in-plane flow patterns in two regions: the abdominal aorta and lower extremity peripheral arteries.

**Methods:**

We performed an institutional review board-approved study in healthy subjects and patients. In 7 healthy subjects, in-plane flow was imaged at 4 stations ranging from the lower legs to the aorto-iliac bifurcation (junction of the distal thigh and upper calf, mid-thigh, junction of the upper thigh and pelvis, upper pelvis). In 5 healthy subjects and 6 patients without abdominal aortopathy, images were acquired through the suprarenal abdominal aorta. Ten patients with known peripheral arterial disease and two patients with stable disease of the abdominal aorta were also evaluated. Peak velocity was compared at each of the 4 stations for cine fast interrupted steady-state in combination with arterial spin labeling and two-dimensional cine phase contrast in patients with normal vessels.

**Results:**

In-plane flow patterns were well visualized in all peripheral arterial stations and in the abdominal aorta, providing a high quality display of hemodynamic patterns along extensive lengths of the vessels. There was very strong positive correlation (*r* = 0.952, *P* < 0.05) and excellent agreement (intraclass correlation coefficient, 0.935; 95% confidence interval, 0.812–0.972) between peak flow velocities measured by cine fast interrupted steady-state in combination with arterial spin labeling and two-dimensional cine phase contrast. In 10 patients with peripheral artery disease and 2 patients with aortic pathology, cine fast interrupted steady-state in combination with arterial spin labeling provided a visual demonstration of abnormal hemodynamics.

**Conclusion:**

This feasibility study suggests that cine fast interrupted steady-state in combination with arterial spin labeling provides an efficient, high quality and physiologically accurate display of in-plane flow patterns over extensive lengths of the lower extremity peripheral arteries, which can be difficult to achieve using other MRA techniques.

**Electronic supplementary material:**

The online version of this article (10.1186/s12968-019-0562-3) contains supplementary material, which is available to authorized users.

## Background

Peripheral artery disease (PAD) is a major health issue worldwide, affecting approximately 202 million people globally [1]. Imaging evaluation for those under consideration for revascularization was traditionally performed using catheter-directed digital subtraction angiography (DSA) [2]. In efforts to reduce the invasiveness and risk, physicians have turned to contrast-enhanced magnetic resonance angiography (CEMRA) and computed tomography angiography (CTA) for non-invasive vascular evaluation [3–5]. CTA is a static technique that provides little information about the hemodynamic status of the arteries, whereas time-resolved CEMRA can be used to provide a dynamic display of arterial flow patterns. However, time-resolved CEMRA has limited temporal resolution and primarily provides qualitative rather than quantitative evaluation of blood flow [6, 7]. Both CTA and time-resolved CEMRA require the administration of contrast agents, which may be contraindicated in patients with impaired renal function.

Cine fast interrupted steady-state in combination with arterial spin labeling (cFASL) is a recently-described nonenhanced MRA technique that provides a DSA-like display of in-plane flow patterns with high temporal resolution [8]. Rather than measure phase shifts that have been created by the flow of arterial spins through magnetic field gradients, which is the underlying principle for phase contrast methods, cFASL quantifies flow velocity based on measuring the temporally-resolved displacement of a radiofrequency (RF)-labeled bolus of arterial spins. Unlike 2-dimensional phase contrast (2DPC), cFASL is insensitive to the effects of partial volume averaging and enables in-plane flow patterns to be directly visualized. We hypothesized that cFASL could be used to efficiently display arterial hemodynamics over extensive lengths of the aorta and lower extremity peripheral arteries.

## Methods

### Subjects

This study was compliant with the Health Insurance Portability and Accountability Act. Four groups of subjects were studied. In the first group cFASL and 2DPC were acquired at four stations throughout the lower extremity arteries in 7 healthy subjects without a history of PAD (4 male, 25–55 years). In the second group, cFASL and 2DPC were acquired through the suprarenal segment of the abdominal aorta in 5 healthy subjects and 6 patients (5 male, 25–75 years) during a clinically-indicated magnetic resonance (MR) exam for pathology outside of the supra-renal aorta. A third group included 10 patients with known peripheral artery pathology (5 male, 46–81 years). A fourth group of 2 patients with disease of the abdominal aorta included one with a chronic type B dissection and one with arcuate ligament syndrome (1 male, 46–48 years). All patients provided written, informed consent except for patients in group 2, who had IRB-approved waiver of consent.

### Imaging protocol

All studies were performed on a 1.5 T system (MAGNETOM Avanto, Siemens Healthineers, Erlangen, Germany) with a standard phased array body coil. No intravenous contrast was administered.

### Localization

In the lower extremity patients and volunteers, standard localizer scans were followed by nonenhanced, electrocardiogram (ECG)-gated quiescent-interval slice-selective (QISS) MRA [9] to provide an anatomical roadmap of the lower-extremity peripheral arteries.

### cFASL

cFASL was used to measure peak flow velocities in healthy vessels and to portray flow patterns in both healthy and diseased arteries. In the first group, imaging in each subject was performed at 4 stations ranging from the lower legs to the aortic bifurcation (junction of the distal thigh and upper calf, mid-thigh, junction of the upper thigh and pelvis, upper pelvis) in a coronal and/or sagittal plane. Several slices were acquired at each station to ensure coverage of the entire thickness of the target vessels. For coronal imaging, 4 to 6 slices (9-mm, 10% overlap) were obtained at each station for bilateral arterial evaluation versus 3 to 4 slices for sagittal imaging (right leg only). In the second group, imaging was performed at a single station in the suprarenal abdominal aorta using 1–3 slices in the sagittal plane. In the third and fourth groups, cFASL was acquired in 1–6 slices in coronal, sagittal and/or axial planes. In some subjects, additional cFASL acquisitions were performed using a thick (e.g. 27-mm) slice to assess the impact of partial volume averaging on image quality.

Spin labeling was performed in all studies using a 10.24-ms duration adiabatic inversion RF pulse which was applied shortly after every second R-wave to inflowing arterial spins over a 25-mm thick region orthogonal to the vessel axis. This process resulted in the interleaved acquisition of labeled and unlabeled data, which helped to minimize misregistration artifact from patient motion. Background suppression was achieved by complex subtraction of the labeled and unlabeled cine images. Imaging parameters included 240 radial views with equidistant azimuthal view angle increment of ~ 15 degrees, 40 shots, sampling bandwidth = 888 Hz/pixel, TE ~ 1.5 ms, echo spacing ~ 3.0 ms, excitation flip angle ~ 60 degrees, prospective cine acquisition, 6 balanced steady-state free precession (bSSFP) repetitions per fast interrupted steady state (FISS) module, matrix 240 × 240 (sagittal) or 300 × 300 (coronal), 27 reconstructed cine phases (no view sharing) for an acquisition window of 500 ms, temporal resolution ~ 20 ms.

### 2D phase contrast

Through-plane 2DPC was performed immediately following cFASL at a single level within each station, with the slice oriented orthogonal to the artery in normal vessels. Based on empirical testing, the initial velocity encoding (VENC) values were 50 cm/s for calf-thigh, 80 cm/s for mid-thigh, 100 cm/s for thigh-pelvis, 120 cm/s for upper pelvis and 150 cm/s for the suprarenal aorta. In the event of aliasing, the VENC was increased by approximately 50% and the 2DPC acquisition was repeated. Other imaging parameters included TE 3.5–5 ms, repetition time 25–50 ms, matrix ~ 244 × 300 and a flip angle of ~ 10 degrees.

### Flow quantification

An ImageJ (National Institutes of Health and the Laboratory for Optical and Computational Instrumentation, University of Wisconsin, Madison, Wisconsin, USA) script was developed to semi-automate calculation of peak velocity from the cFASL in normal vessels. The cine images were uploaded and a maximum intensity projection (MIP) image was created including all the slices. A spline path was manually drawn along the course of the vessel and the program calculated the peak velocity based on frame-to-frame displacement of the bolus along the manually drawn vessel course and the time resolution between successive cine frames. Bolus arrival occurred when the signal intensity of the vessel exceeded the threshold value of S = μ_B_ + 2σ_B_, where μ_B_ and σ_B_ are the mean and standard deviations of a nearby circular region of background signal (size ≈300mm^2^). Peak velocity on cFASL was computed as the maximal value of the distance of bolus travel in one frame divided by the time resolution of the frame. The peak velocities were calculated individually by two radiologists and any discrepancies were reviewed with agreement in consensus by two radiologists (E.A., R.R.E.). Peak velocities from through-plane 2DPC were calculated using Argus Flow (Siemens Healthineers).

### Image quality analysis

Image quality for all normal and diseased cases was assessed individually by two radiologists (E.A. and R.R.E.) based on anonymized MIP images. Thirty-nine images of healthy subjects or normal patients were evaluated and 12 images of pathologic segments. The following 4-point scoring system was used: 1 = non-diagnostic, image quality inadequate for diagnosis; 2 = fair, image quality marginally acceptable for diagnosis; 3 = good, image quality adequate for confident diagnosis; and 4 = excellent, excellent image quality providing a highly-confident diagnosis.

### Statistical analysis

The relationships between the measurements acquired in normal vessels using both 2DPC and cFASL were examined with the Pearson product-moment correlation coefficient analysis. Two-way mixed-effects single measures intra-class correlation coefficient (ICC) for absolute agreement was used to assess agreement between flow velocity measured on 2DPC and cFASL using IBM SPSS Statistics for Windows, version 22 (Statistical Package for the Social Sciences (SSPS) International Business Machines, Inc., Armonk, New York, USA). Agreement was also evaluated using the approach by Bland-Altman [10] by calculating the mean difference (μ) and standard deviation (SD) of the difference in flow velocity measurements between 2DPC and cFASL from all subjects and analysis planes. From these data, the 95% limits of agreement (μ ± 1.96SD) were calculated. For image quality, the mean score and standard deviation for each reviewer overall, in normal segments and in diseased segments was calculated.

## Results

Scan time for the cFASL sequence was approximately 65 s per slice. cFASL of each vessel segment was typically acquired in 1–6 slices depending on the location and plane of imaging, which gives an overall scan time of 65–390 s. For unilateral evaluation, sagittal cFASL was more efficient than coronal imaging as fewer slices were needed to encompass the arteries, whereas imaging in the coronal plane permitted direct comparison of flow between limbs. In one subject, 2DPC through the upper thigh was not evaluable due to susceptibility artifact, so neither 2DPC nor cFASL data at this level was included in the analysis. Post-processing time was rapid (approximately 1 to 2 min per level) for both 2DPC and cFASL.

### Peak velocity measurements in Normal vessels overall

**Fig. 1:**
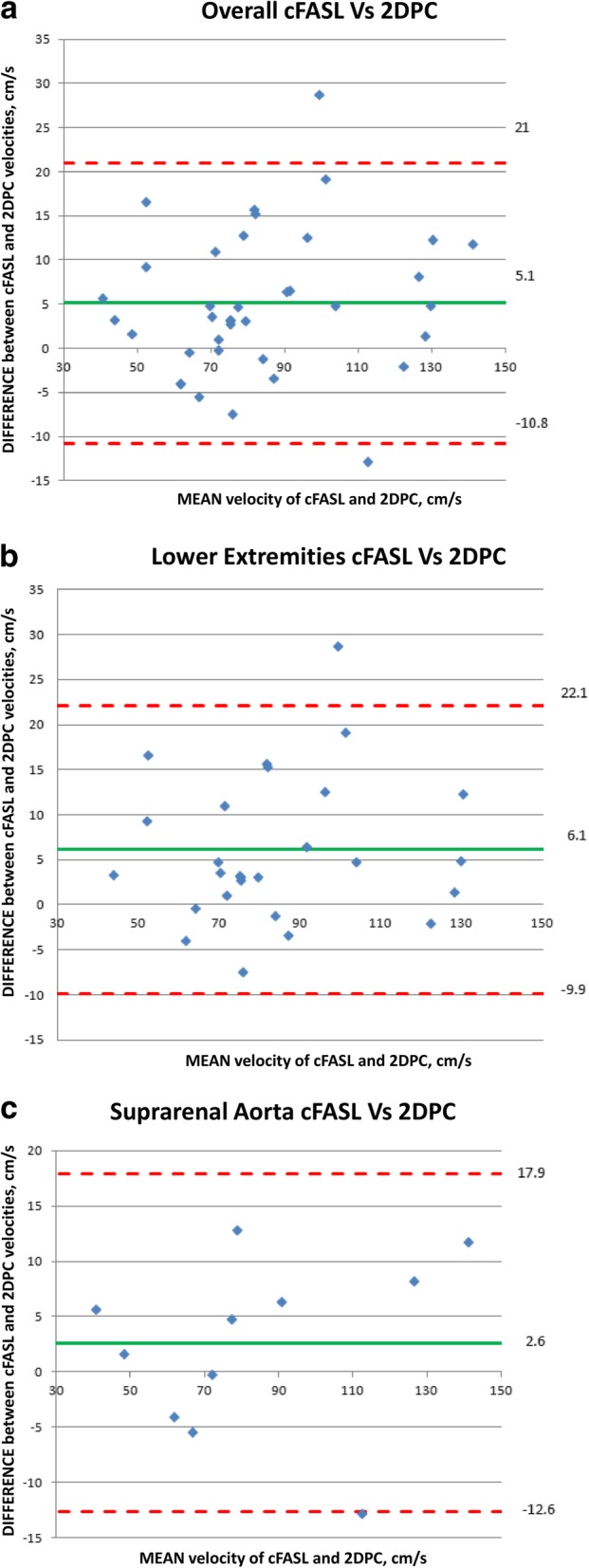
**a** Bland-Altman diagram showing the plot of the difference between the flow velocity measured by cine fast interrupted steady state in combination with arterial spin labeling (cFASL) and 2D phase contrast (PC) at all stations in both cohorts against the mean velocity of the pair (*n* = 38). Red lines show the 95% limits of agreement, and the green line shows the mean value of the differences. **b** Bland-Altman diagram showing the plot of the difference between the flow velocity measured by cFASL and 2DPC at all stations in the lower extremity volunteers against the mean velocity of the pair (*n* = 27). Red lines show the 95% limits of agreement, and the green line shows the mean value of the differences. **c** Bland-Altman diagram showing the plot of the difference between the flow velocity measured by cFASL and 2DPC in the suprarenal aorta against the mean velocity of the pair (*n* = 11). Red lines show the 95% limits of agreement, and the green line shows the mean value of the differences

**Fig. 2:**
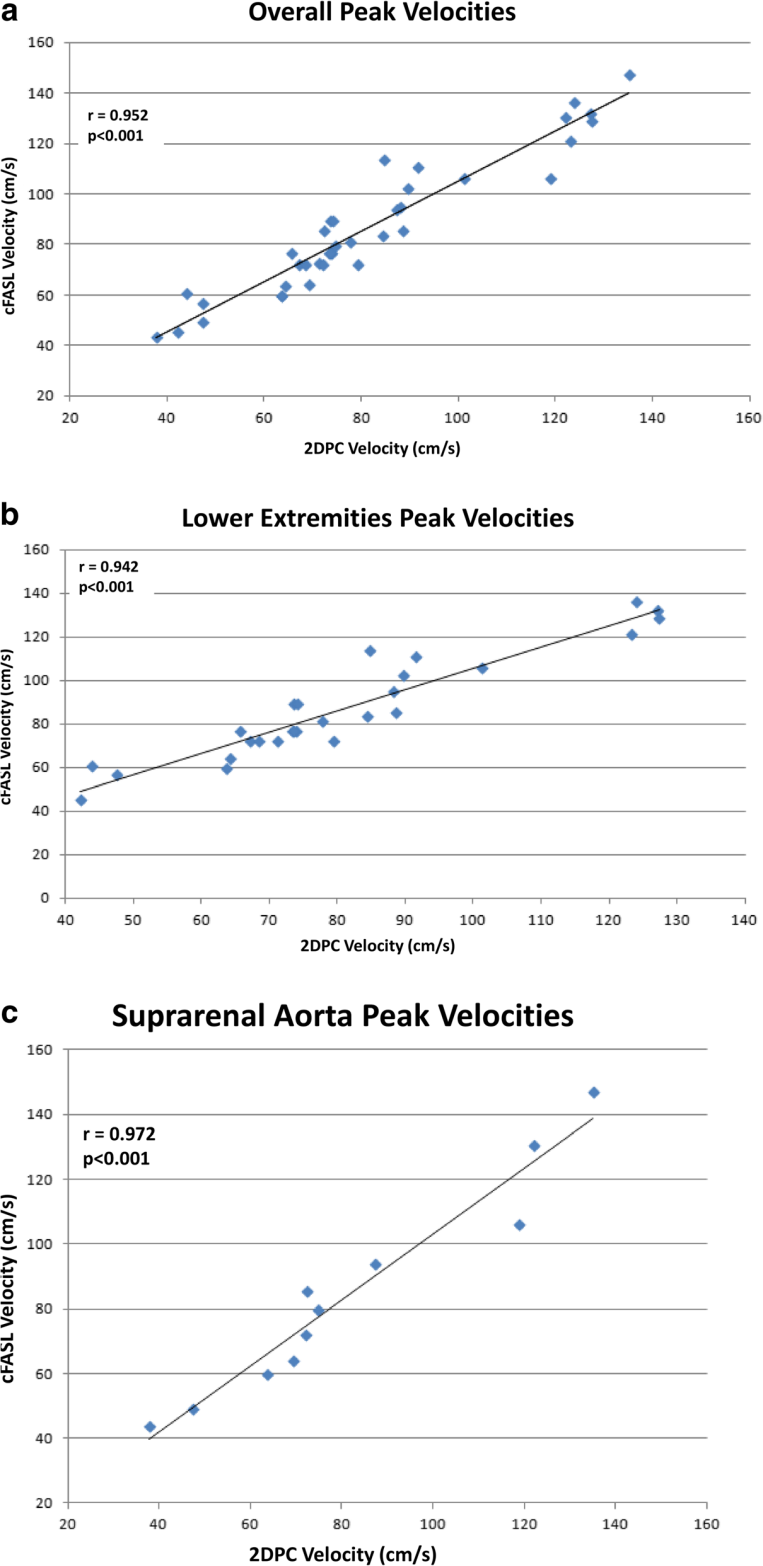
**a** Linear relationship between overall peak flow velocity measurements in the lower extremities and suprarenal aorta using cFASL and 2DPC. **b** Linear relationship between lower extremity peak flow velocity measurements using cFASL and 2DPC. **c** Linear relationship between suprarenal aorta peak flow velocity measurements using cFASL and 2DPC

For cFASL peak velocity measurements in normal vessels, the mean, σ and range were 86.4, 26.5, 43.5–147.0 cm/s; for 2DPC, the mean, SD and range were 81.3, 25.4, 37.8–135.2 cm/s. Bland-Altman analysis of agreement of cFASL and 2DPC measurements at all levels revealed a mean velocity difference of 5.1 cm/s (SD= 7.8; 95% limits of agreement -10.8/21.0 cm/s) (Fig. 1a). 2DPC and cFASL peak velocity measurements were found to be in excellent agreement (ICC = 0.935, 95% confidence interval: 0.812–0.972, *P* < 0.001) and were also strongly correlated (*r* = 0.952, *P* < 0.001) (Fig. 2a).

cFASL velocity measurements in normal vessels overall including both the lower extremities and suprarenal aorta were greater than 2DPC at 28/38 (74%) individual levels with mean peak velocities of 86.5 cm/s and 81.4 cm/s respectively.

### Peak velocity measurements healthy subjects lower extremities

For cFASL in the lower extremities including all levels, the mean, SD, and range of peak velocities were 87.3, 24.3, 45.5–136.3 cm/s; for 2DPC, the mean, SD, and range were 81.2, 23.4, 42.2–127.4 cm/s (Table 1). Bland-Altman analysis of agreement of cFASL and 2DPC measurements at all levels revealed a mean velocity difference of 6.1 cm/s (SD = 8.15; 95% limits of agreement -9.9/22.1 cm/s) (Fig. 1b). Peak velocity measurements acquired using both techniques were strongly correlated (*r* = 0.942, *P* < 0.001) (Fig. 2b). ICC showed excellent agreement overall and at each station individually, except in the proximal thigh where agreement was good (Table 2).

**Table 1:** Peripheral artery flow measurements

Volunteers	1	2	3	4	5	6	7
Calf-Thigh							
2DPC	63.7 cm/s	47.5 cm/s	67.2 cm/s	89.7 cm/s	44.0 cm/s	73.5 cm/s	42.2 cm/s
cFASL	59.7 cm/s	56.8 cm/s	72 cm/s	102.3 cm/s	60.6 cm/s	76.7 cm/s	45.5 cm/s
Mid-Thigh							
2DPC	73.7 cm/s	64.3 cm/s	88.2 cm/s	101.3 cm/s	71.3 cm/s	124.0 cm/s	77.9 cm/s
cFASL	89.4 cm/s	63.9 cm/s	94.7 cm/s	106.1 cm/s	72.4 cm/s	136.3 cm/s	81.0 cm/s
Proximal Thigh							
2DPC	84.5 cm/s	65.7 cm/s	74.0 cm/s	84.8 cm/s	68.4 cm/s	123.2 cm/s	^a^
cFASL	83.3 cm/s	76.7 cm/s	76.7 cm/s	113.6 cm/s	72.0 cm/s	121.2 cm/s	^a^
Pelvis							
2DPC	91.6 cm/s	79.4 cm/s	88.6 cm/s	127.2 cm/s	73.6 cm/s	127.4 cm/s	74.2 cm/s
cFASL	110.8 cm/s	72.0 cm/s	85.2 cm/s	132.1 cm/s	76.7 cm/s	128.8 cm/s	89.5 cm/s

^a^Values excluded due to technical issue with acquisition of 2DPC at this level

Flow measurements at each level of the lower extremities in cm/s calculated from analysis of 2D phase contrast (PC) and cine fast interrupted steady-state in combination with arterial spin labeling (cFASL) data in seven healthy subjects

**Table 2:** Intraclass correlation coefficients (ICC) of velocities measured by 2DPC and cFASL

	ICC	95% CI	Significance
All levels	0.913	0.681, 0.968	< 0.001
Calf-Thigh	0.878	0.272, 0.979	< 0.001
Mid-Thigh	0.935	0.419, 0.990	< 0.001
Proximal Thigh	0.824	0.265, 0.973	0.008
Pelvis	0.913	0.619, 0.984	0.001

### Peak velocity measurements patients – suprarenal aorta

For cFASL in the suprarenal aorta, the mean, SD and range were 84.6, 32.7, 43.5–147 cm/s; for 2DPC, the mean, SD and range were 81.9, 31.0, 37.8–135.2 cm/s (Table 3). Bland-Altman analysis of reproducibility of cFASL and 2DPC measurements at all levels revealed a mean velocity difference of 2.6 cm/s (SD = 7.8; 95% limits of agreement -12.6/17.9 cm/s) (Fig. 1c). Peak velocity measurements acquired using both techniques were found to be in excellent agreement (ICC = 0.970, 95% confidence interval: 0.897–0.992, *P* < 0.001) and were strongly correlated (*r* = 0.972, *P* < 0.001) (Fig. 2c).

**Table 3:** Suprarenal aorta flow measurements

Suprarenal Aorta	1 cm/s	2 cm/s	3 cm/s	4 cm/s	4 cm/s	6 cm/s	7 cm/s	8 cm/s	9 cm/s	10 cm/s	11 cm/s	12 cm/s
2DPC	72.6	72.2	122.2	37.8	87.4	135.2	47.5	63.7	118.9	74.77	72.4	69.3
cFASL	64.4	72	130.4	43.5	93.8	147	49.2	59.7	106.1	79.54	85.2	63.9

Flow measurements in the suprarenal aorta in cm/s calculated from analysis of 2DPC and cFASL data in twelve patients

### Evaluation of flow patterns in healthy vessels and vascular pathology

**Fig. 3:**
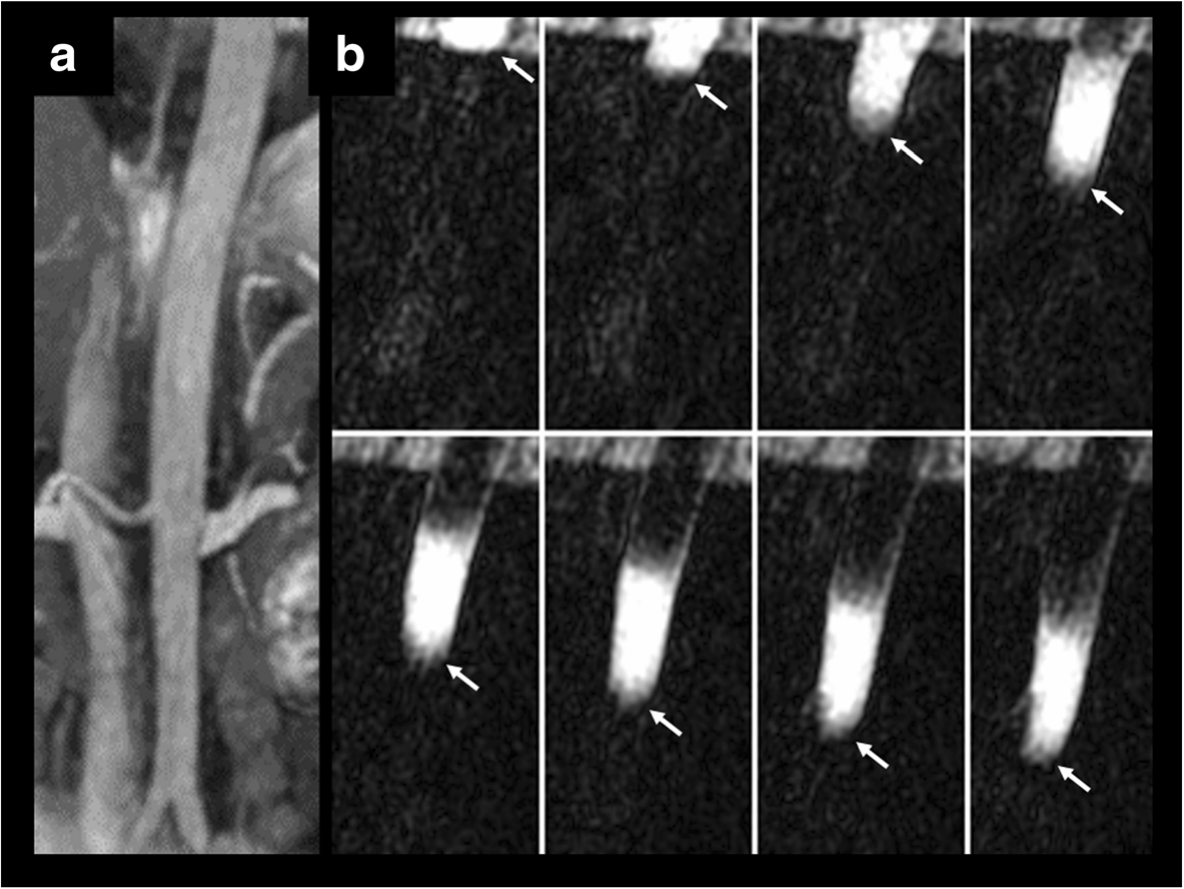
54-year-old female healthy subject. **a** Scout coronal bSSFP image through the abdominal aorta. **b** Sagittal cFASL shows progression of the labeled bolus (arrows) through the upper abdominal aorta (8 of 32 frames shown, temporal resolution = 20 ms). The labeled bolus is plug-shaped as opposed to the parabolic shape seen with laminar flow in the smaller peripheral arteries

**Fig. 4:**
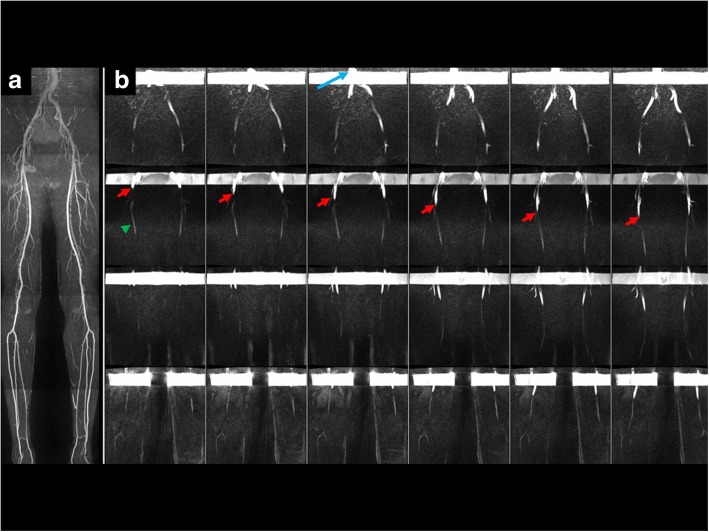
55-year-old healthy male. **a** QISS MRA shows normal appearance of the peripheral arteries. **b** Coronal cFASL (maximum intensity projections across all slices, 6 of 22 frames shown) acquired through the upper pelvis, thigh-pelvis junction, mid-thigh and calf-thigh junction (top row to bottom row, respectively). Symmetrical progression of the labeled bolus (red arrows) through the legs is observed. A small amount of arterial signal can be observed in early frames (green arrowhead), which represents residual signal from tagging that occurred in a prior cardiac cycle. Retrograde flow in veins is observed above each tag (long blue arrow = inferior vena cava)

Using cFASL, in-plane flow patterns were visualized in all evaluated peripheral arterial segments (Fig. 3) and in the abdominal aorta (Fig. 4). Additional movie files show this in more detail [see Additional files 1 and 2]. Hemodynamic effects of vascular pathology including dissection, stenosis and occlusion were also well depicted.

**Fig. 5:**
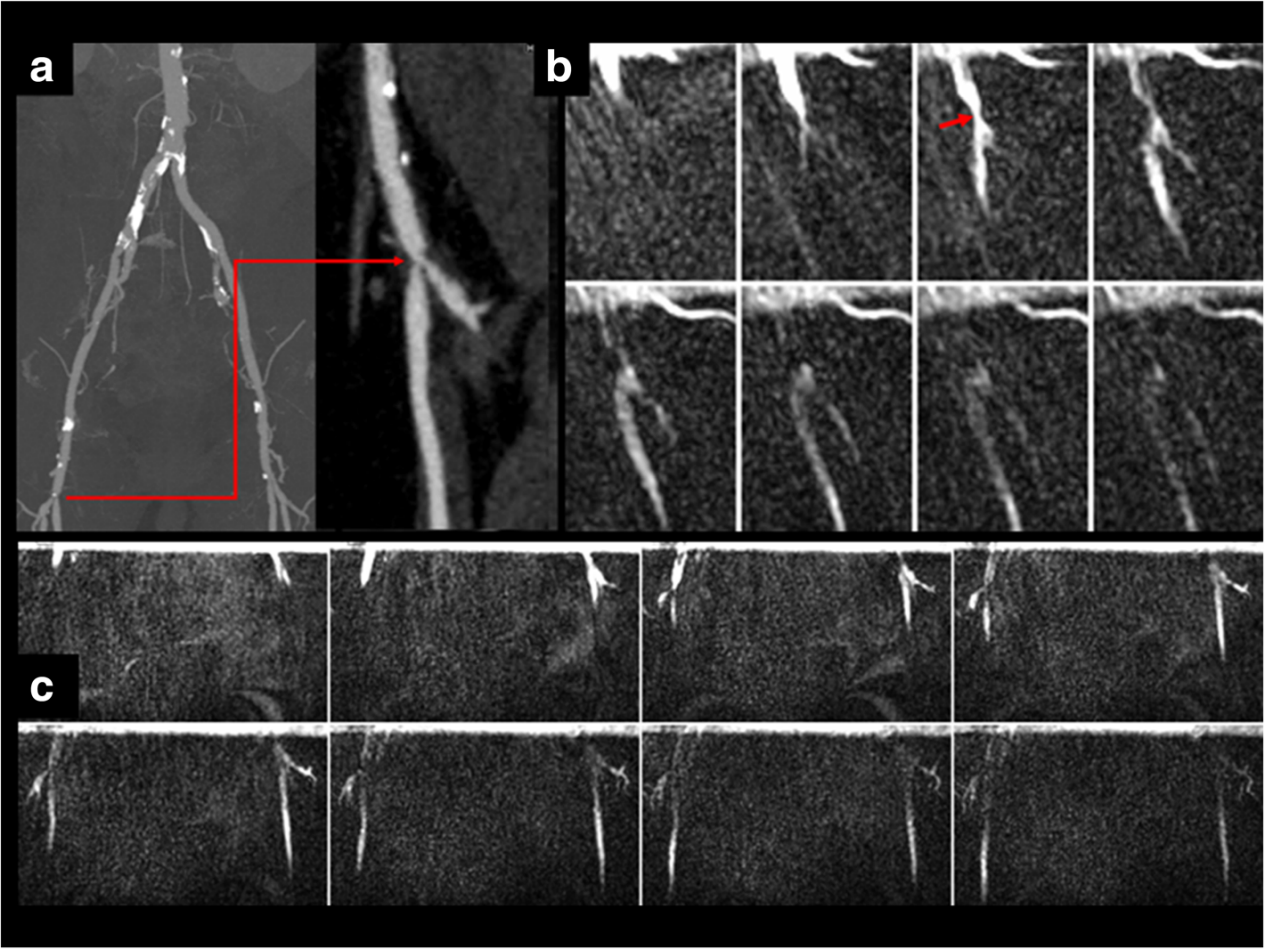
58-year-old female who presented with disabling right leg pain. **a** computed tomography angiography (CTA) shows moderate stenosis at the bifurcation of the right common femoral artery, best shown in the magnified sagittal multi-planar reconstruction. **b** Sagittal cFASL (8 of 32 frames shown) shows the stenosis which appears comparable in severity to the CTA. **c** Coronal cFASL illustrates a slight delay in the progression of the labeled bolus in the right common iliac and superficial femoral arteries (most apparent in the first few frames) with respect to the contra-lateral arteries, consistent with a hemodynamically-significant lesion

**Fig. 6:**
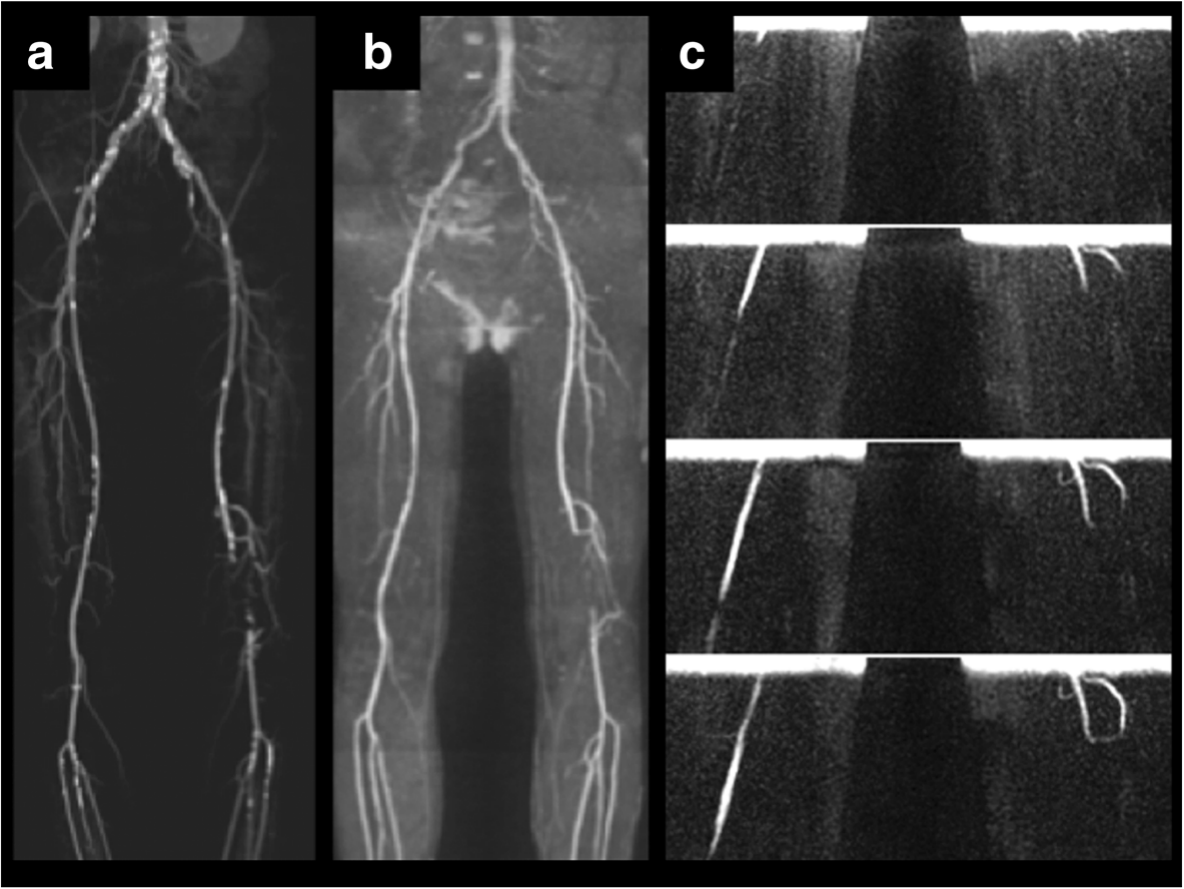
67-year-old male with history of left leg pain. CTA (**a**) and QISS MRA (**b**) show occlusion of the distal left superficial femoral artery (SFA) with distal reconstitution through collateral vessels. **c** Coronal cFASL shows normal, rapid progression of the labeled bolus through the right SFA, in contrast to the markedly delayed progression of the labeled bolus through the left-sided collaterals distal to the occlusion

**Fig. 7:**
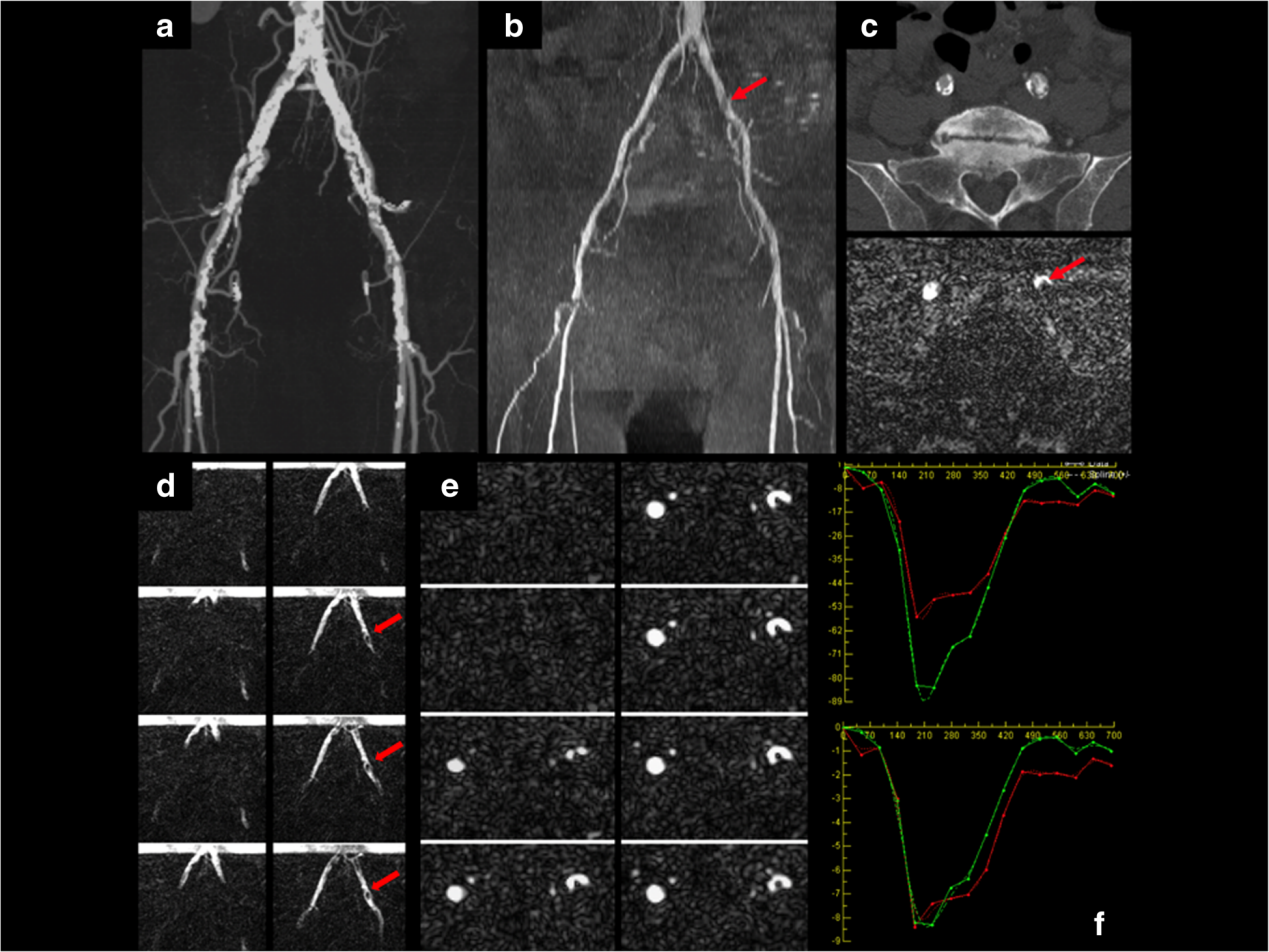
69-year-old male with right leg claudication secondary to occlusion of the proximal right superficial femoral artery. **a** CTA demonstrates heavily calcified atherosclerotic disease. **b** QISS MRA. Red arrow = incidental moderate stenosis of the left common iliac artery (CIA). **c** Axial slice from CTA (top) and QISS MRA (bottom) shows the left CIA stenosis. **d** Coronal cFASL shows a similar rate of progression of the labeled bolus on the two sides despite the presence of the left-sided stenosis (red arrows). This finding is suggestive that the stenosis is not hemodynamically significant. The left CIA plaque is visible as a partial filling defect in the labeled bolus of arterial blood. **e** Thin-slice (3-mm) axial cFASL at the level of the stenosis shows the arterial lumen (white) in high contrast to the plaque (dark). **f** Axial 2DPC at the level of the stenosis shows flow velocity (top) and bulk flow (bottom) for the right (red) and left (green) vessels. 2DPC confirms that the stenosis is not hemodynamically significant since bulk flow is approximately equal between the two sides during the systolic phase of the cardiac cycle, despite the increased peak flow velocity within the stenosis

**Fig. 8:**
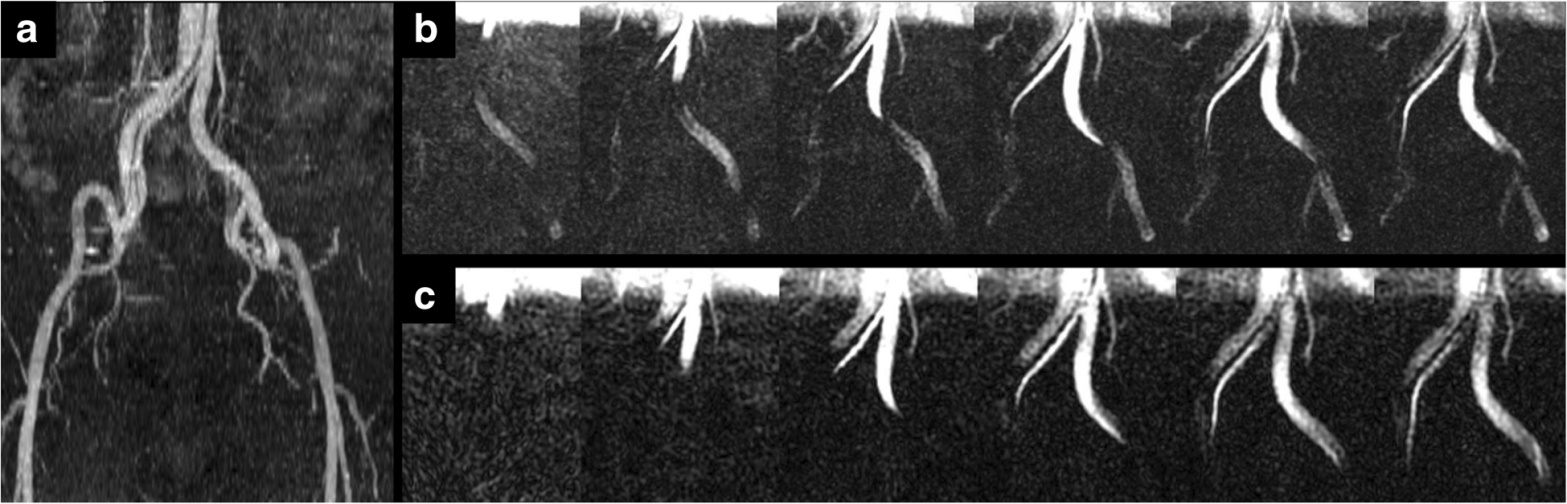
46-year-old male with a stable type B aortic dissection under surveillance. **a** Maximum intensity projection from QISS MRA shows the extension of the aortic dissection into the right common iliac artery. **b** Maximum intensity projection of five 9-mm thick slices using cFASL shows faster progression of the labeled bolus through the narrow, medially-located true lumen compared to the wider false lumen. **c** Single 27-mm slice using cFASL shows similar findings to (**b**) in one-fifth the scan time of the multi-slice cFASL acquisition

Results of studies in patients with vascular pathology are summarized in Table 4 and examples are provided in Figs. 5, 6, 7 and 8.

**Table 4:** Results of patients with vascular pathology

Group 3 - Peripheral Artery Pathology						
	Age	Sex	Pathology	Imaging Plane	Slices/level	Qualitative Imaging Findings
Patient 1	58	F	Right SFA Stenosis	Coronal, Sagittal	3	Hemodynamically significant stenosis
Patient 2	67	M	Left SFA Occlusion	Coronal	3–5	Collateral vessels on the left with slower flow compared to right SFA
Patient 3	69	M	Left CIA Stenosis	Coronal	4–5	Appeared high grade on static CT images but flow was similar bilaterally at rest and the patient was not symptomatic on the left side
Patient 4	73	F	Fem-pop Graft Anastomosis Stenosis	Coronal, Sagittal	3	Hemodynamically significant stenosis with slower flow above the stenosis and rapid flow through the stenosis
Patient 5	74	F	Mid Fem-pop Graft Stenosis	Coronal, Sagittal	1	Symmetric flow in bilateral grafts suggests the stenosis is not hemodynamically significant at rest
Patient 6	76	M	Left CFA Stenosis	Coronal	3,7	Decreased flow on the left side compared to the right side consistent with a hemodynamically significant stenosis
Patient 7	81	F	Right SFA Occlusion	Coronal	5	Collateral thigh vessels with reduced flow compared to the left side
Patient 8	78	F	Multifocal Below-knee Occlusive Atheroma	Coronal	5	Markedly reduced flow below the knee bilaterally with collateral vessels seen
Patient 9	65	M	Right CFA Stenosis	Coronal	5	Tortuous vessel but the stenosis was not hemodynamically significant
Patient 10^a^	46	M	Right CIA/EIA Dissection	Coronal	5, 1	Differential flow patterns in true and false lumen
Group 4 - Abdominal Aorta Pathology						
	Age	Sex	Pathology			Qualitative Imaging Findings
Patient 1	48	F	Median Arcuate Ligament Syndrome	Sagittal, Sagittal oblique	1–2	Severe stenosis of the celiac axis by the median arcuate ligament with reduced flow in the celiac axis
Patient 2^a^	46	M	Chronic dissection	Coronal	6	Differential flow patterns in true and false lumen

^a^Patient 10 in group 3 and patient 2 in group 4 are the same patient but imaged at different levels as pathology was seen in both areas of interest

For instance, Fig. 5 demonstrates a focal stenosis of the origin of the right SFA (Patient 1). The stenosis was visually confirmed on cFASL and differential in-plane flow was noted between the right and left sides consistent with a hemodynamically-significant lesion. Additional movie files show this finding in more detail [see Additional file 3]. A hemodynamically-significant lesion was confirmed on 2DPC which measured peak velocities of 87.9, 158.2 and 66.8 cm/s proximal to, within, and distal to the stenosis.

Figure 8 is from a subject with a chronic type B aortic dissection extending from the thoracic aorta distally through the abdominal aorta and into the right common iliac artery (Patient 10). Maximum intensity projection of five coronal 9-mm slices using cFASL (Fig. 8b) displayed markedly different flow patterns in the true and false lumens. A single 27-mm cFASL slice provided a comparable display of flow patterns (Fig. 8c) to the multi-slice cFASL acquisition in a fraction of the scan time. Additional movie files show this finding in more detail [see Additional file 4].

### Image quality

Mean (± SD) overall, normal and diseased segment scores were 3.6 (±0.5), 3.7 (±0.5) and 3.2 (±0.4), respectively, for reader 1 and 3.4 (±0.7), 3.5 (±0.6) and 3.1 (±0.8), respectively, for reader 2. These results are compatible with overall good image quality in both normal and diseased segments, although image quality was slightly better in normal segments.

## Discussion

This preliminary study demonstrates that cFASL provides a physiologically accurate display of in-plane flow patterns over extensive lengths of the lower extremity peripheral arteries and abdominal aorta. The FISS readout which is key to this technique is a novel variant of the widely-used bSSFP pulse sequence. It combines a frequently-interrupted echo train with a radial k-space trajectory, while periodic application of gradient and radiofrequency spoiling suppresses flow artifacts from out-of-slice and off-resonant spins [11]. FISS builds upon prior work using interrupted steady-state sequences, such as the S^5^FP technique [12]. With this earlier technique, which utilized a Cartesian k-space trajectory, the frequent disruption of the steady-state signal could produce severe image artifacts. To avoid these artifacts, FISS uses a radial k-space trajectory with equidistant view angle increments. The benefit is decreased flow artifact and fat signal compared to traditional bSSFP, with greatly reduced flow saturation effects compared with fast low-angle shot (FLASH) [11]. The combination of FISS with ASL was recently described to enable the visualization of in-plane flow patterns within the major arteries of the chest and abdomen [8]. However, validation of cFASL for the measurement of in vivo flow velocities has not previously been described, nor has it been used to image vascular pathology involving the peripheral arteries or abdominal aorta.

In this study, both large and small vessels were evaluated with cFASL. In healthy subjects, the suprarenal abdominal aorta has a predominantly plug flow pattern whereas the smaller peripheral arteries have a predominantly laminar flow pattern [13]. In individuals without significant arterial pathology, we found that peak velocity measurements using cFASL correlated well with 2DPC for both plug and laminar flow patterns. This finding suggests that cFASL may be applicable for flow measurements in both large and small arteries.

For *qualitative* evaluation, cFASL reliably depicted in-plane flow patterns in both healthy and diseased vessels. For instance, visual analysis of time-resolved cFASL images in PAD patientsreadily demonstrated acceleration of the labeled bolus through hemodynamically-significant stenoses along with differential rates of bolus progression versus healthy vessels in the contralateral limb. Tortuous progression of the labeled bolus through collateral vessels, swirling flow in aneurysms, as well as differential progression of the labeled bolus through the true and false lumens of an aortic dissection were also readily apparent. However, for *quantitative* evaluation of flow velocities in the setting of significant arterial pathology, cFASL has limitations since the labeled bolus must remain substantially intact and trackable over the measurement period. For instance, due to dispersion of the labeled bolus from turbulence, we found that the technique was not well suited for flow measurements across hemodynamically-significant stenoses.

Unlike 2DPC, cFASL is largely insensitive to partial volume averaging due to the extreme level of background signal suppression. For instance, a single 27-mm thick slice allowed the semi-projective depiction of arterial flow patterns through extensive lengths of an aortic dissection despite the small caliber of the true lumen. Using such thick slices, one can image substantial lengths of a peripheral artery using a single cine image series, enabling a several-fold reduction in exam time compared with the predominantly multi-slice approach used in the current study. However, further study is needed to determine whether the accuracy of the flow velocity measurements is maintained with thick slices.

2DPC has been used since the 1980s for evaluation of pulsatile flow in the body [14, 15] and has been validated and applied to the lower extremities since the 1990s [16–18]. While 2DPC allows accurate measurement of through-plane flow, considerations of scan time generally limit its use to a few slice locations. Moreover, it is suboptimal for imaging lower extremity arteries in a non-axial orientation due to partial volume averaging between flowing spins and stationary background tissue, which can cause erroneous velocity measurements.

We found that peak velocities measured by cFASL were consistently higher compared to 2DPC (at 28 of 38 stations). This discrepancy could be explained in part by the fact that, while 2DPC is a well-established technique for through-plane flow quantification [16–18], it has been shown to consistently underestimate velocities compared to Doppler ultrasound [19]. Compared with 2DPC, 4D flow (i.e. 3D cine PC) would have provided a more accurate reference standard for flow quantitation. However, it was not used in the current study due to impractically long scan times (e.g. 10 to 15 min per station) and time-consuming image analysis [20]. Despite the limitations of 2DPC, we found very strong positive correlation and excellent agreement between cFASL and 2DPC for the quantification of peak flow velocities throughout the peripheral arterial territory in healthy vessels.

### Limitations

There are several limitations with the cFASL technique. As with other bSSFP-based imaging techniques, the FISS readout is prone to artifacts from off-resonance effects, e.g. near a hip prosthesis. Imaging of the pelvic vessels and suprarenal aorta was performed during free-breathing with satisfactory image quality in our cohort of subjects. Respiratory compensation might be necessary to avoid motion artifacts in some subjects. In very small vessels with rapid laminar flow, it may be difficult to accurately discern the leading edge of the labeled bolus. Furthermore, while cFASL is well-suited to measuring flow velocities over limited portions of the cardiac cycle, phase contrast techniques are easier to apply when flow measurements are required over the entire cardiac cycle. Finally, the finite temporal resolution of both cFASL and 2DPC may negatively impact the accuracy of peak velocity measurements.

## Conclusion

This feasibility study suggests that cFASL provides an efficient, high quality and physiologically accurate display of in-plane flow patterns over extensive lengths of the abdominal aorta and lower extremity peripheral arteries. Further studies will be required to determine the value of this technique in patients with peripheral arterial disease.

## Additional files

Additional file 1 Dynamic display corresponding to Fig. 1. (PPTX 800 kb)

Additional file 2 Dynamic display corresponding to Fig. 2. (PPTX 8739 kb)

Additional file 3 Dynamic display corresponding to Fig. 6. (PPTX 1341 kb)

Additional file 4 Dynamic display corresponding to Fig. 8c. (PPTX 604 kb)

## Data Availability

The datasets used and/or analyzed during the current study are available from the corresponding author on reasonable request

## Abbreviations

2DPC: 2-dimensional phase contrast
bSSFP: Balanced steady-state free precession
CE: Contrast enhanced
cFASL: cine fast interrupted steady-state in combination with arterial spin labeling
CIA: Common iliac artery
CMR: Cardiovascular magnetic resonance
CTA: Computed tomography angiography
DSA: Digital subtraction angiography
ECG: Electrocardiogram
FISS: Fast interrupted steady state
MIP: Maximal image projection
MRA: Magnetic resonance angiography
PAD: Peripheral arterial disease
PC: Phase contrast
QISS: Quiescent interval slice-selective
RF: Radiofrequency
SFA: Superficial femoral artery
VENC: Velocity encoding
