# Supplementary figures and images for: Dynamic quantitative nonenhanced magnetic resonance angiography of the abdominal aorta and lower extremities using cine fast interrupted steady-state in combination with arterial spin labeling: a feasibility study
Source: J Cardiovasc Magn Reson. 2019 Sep 2;21:55. doi: 10.1186/s12968-019-0562-3 (PMC6717984; doi:10.1186/s12968-019-0562-3)

## Slide 1
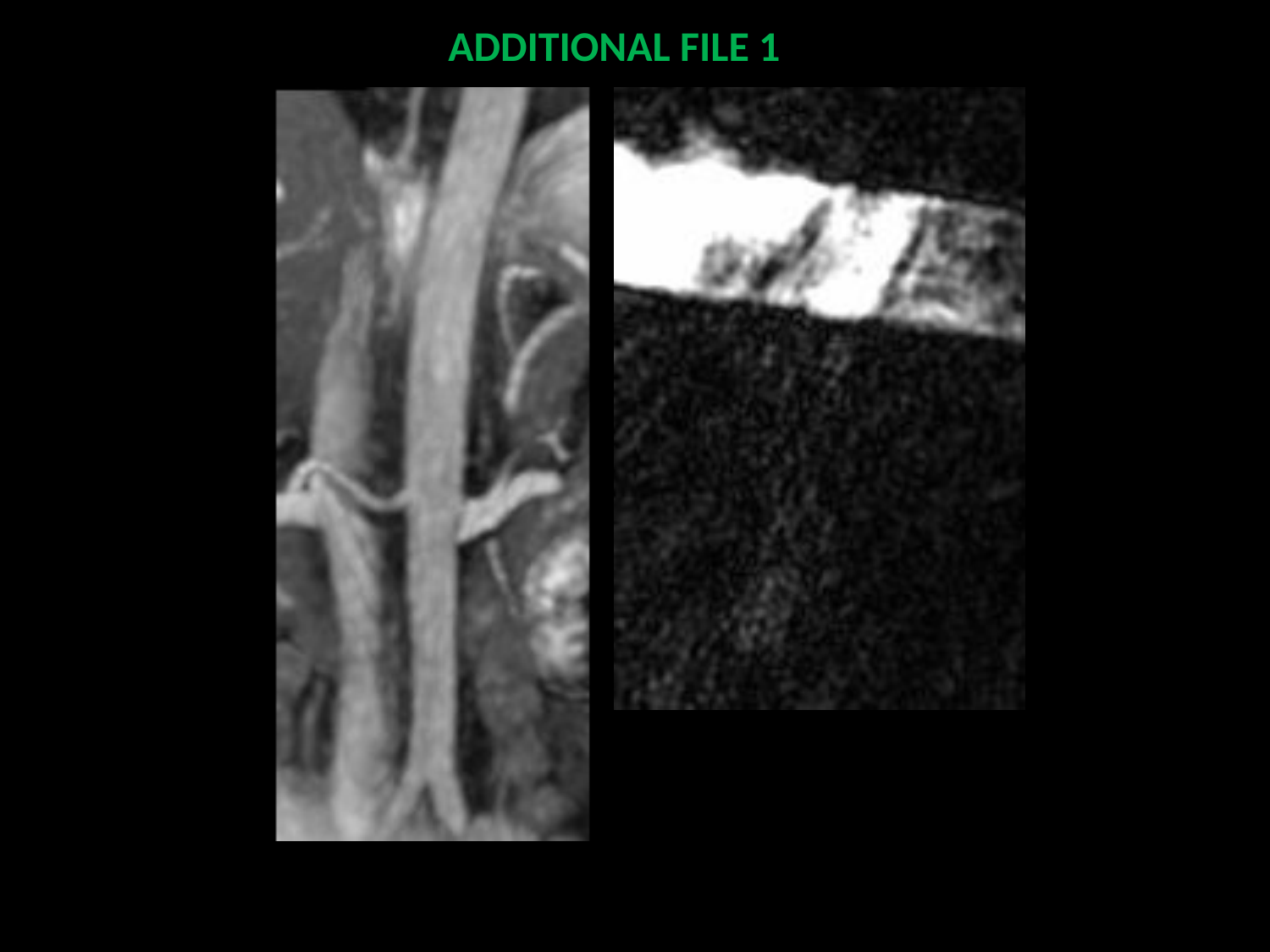

ADDITIONAL FILE 1

Supplement: Supplementary file 1 — Dynamic display corresponding to Fig. 1. (PPTX 800 kb) [file 12968_2019_562_MOESM1_ESM.pptx]

## Slide 1
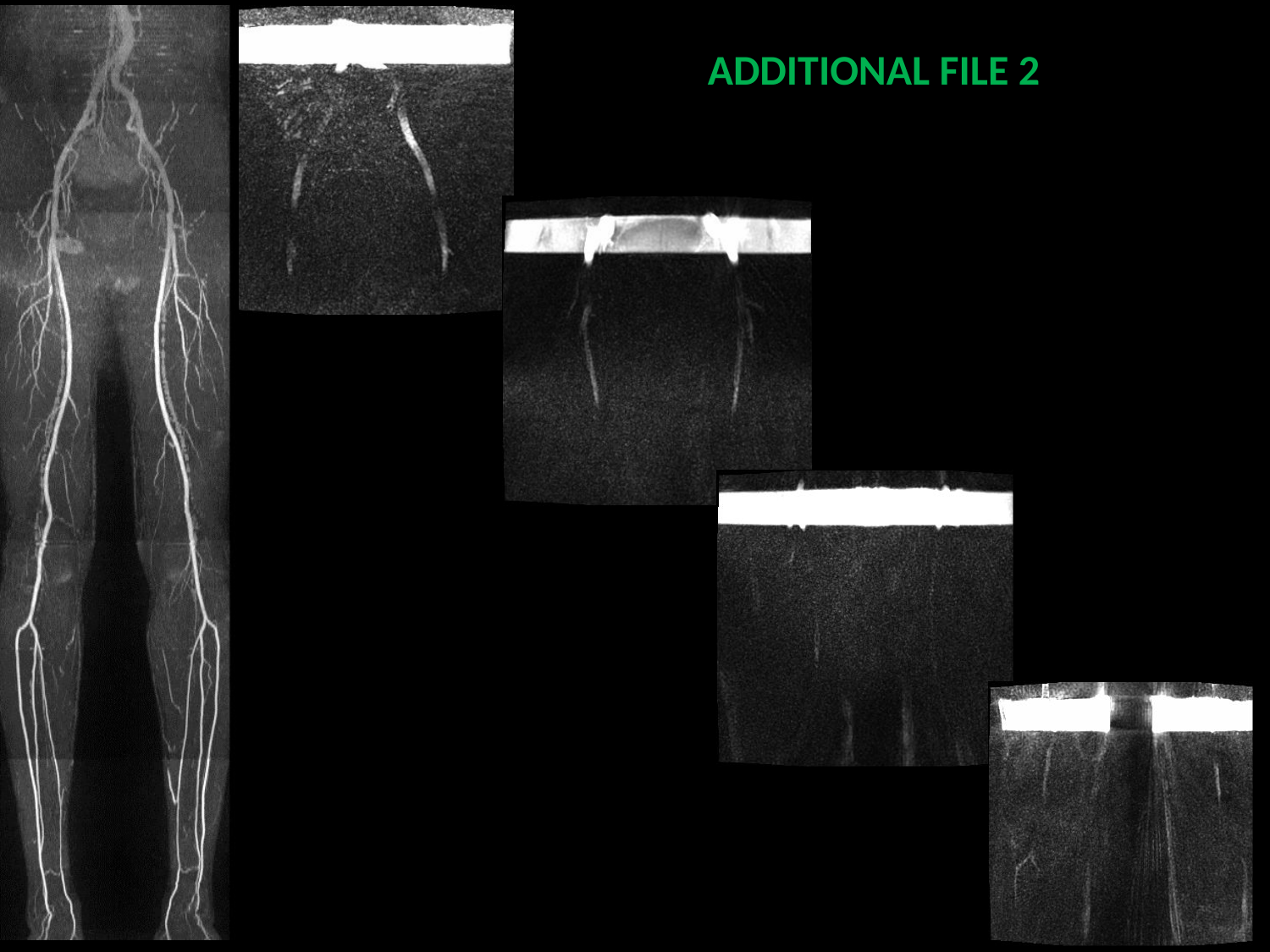

ADDITIONAL FILE 2

Supplement: Supplementary file 2 — Dynamic display corresponding to Fig. 2. (PPTX 8739 kb) [file 12968_2019_562_MOESM2_ESM.pptx]

## Slide 1
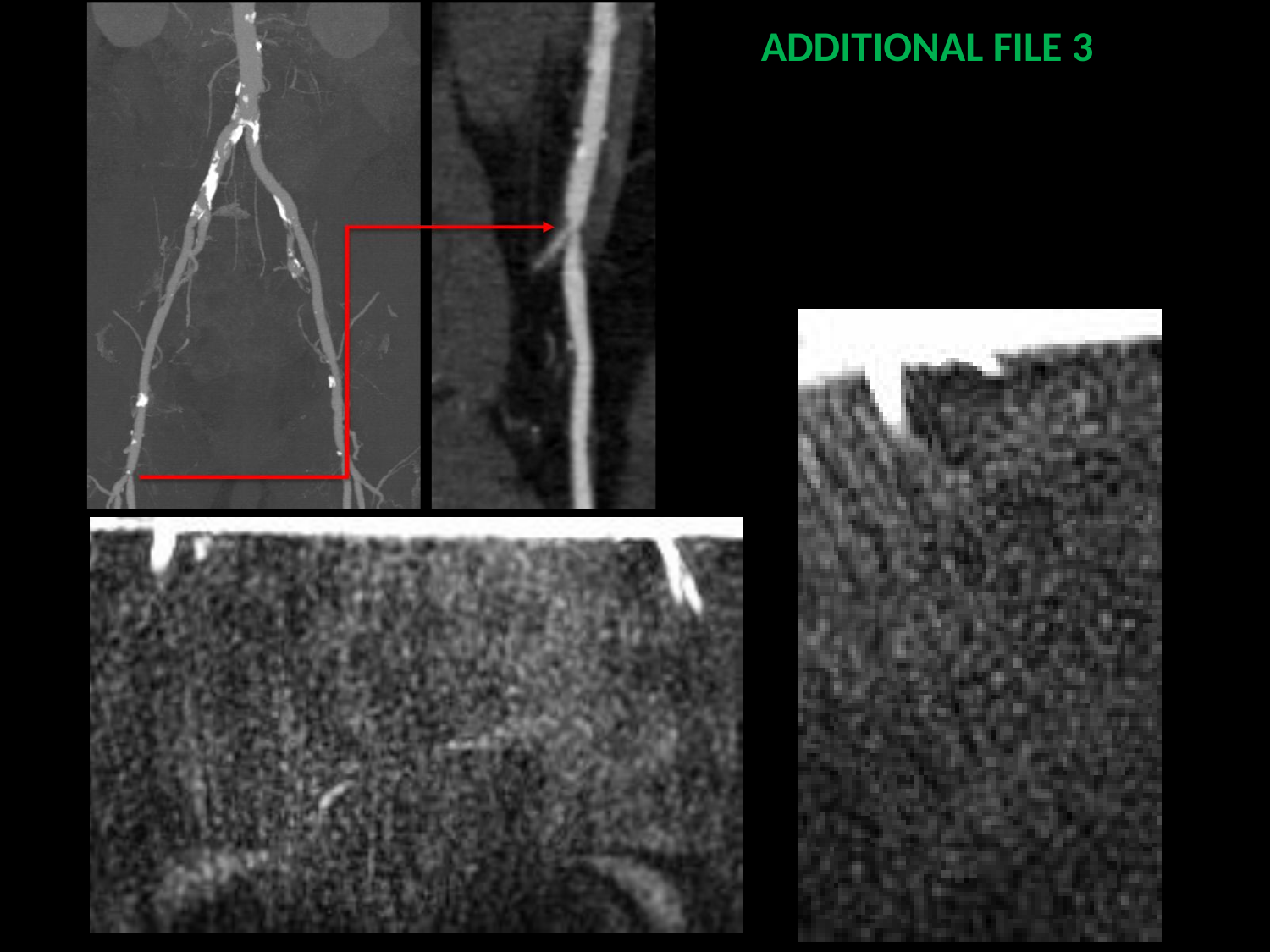

ADDITIONAL FILE 3

Supplement: Supplementary file 3 — Dynamic display corresponding to Fig. 6. (PPTX 1341 kb) [file 12968_2019_562_MOESM3_ESM.pptx]

## Slide 1
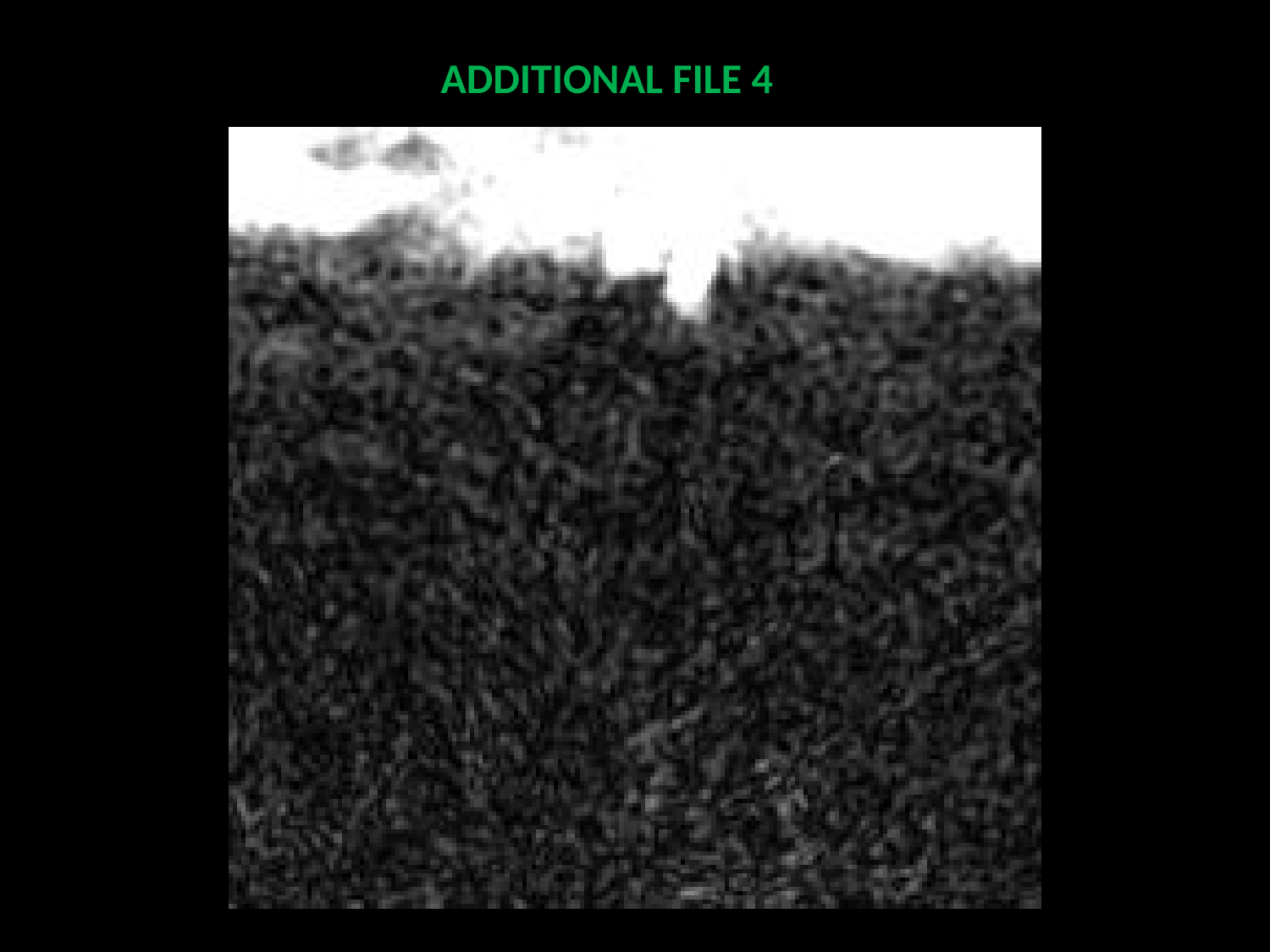

ADDITIONAL FILE 4

Supplement: Supplementary file 4 — Dynamic display corresponding to Fig. 8c. (PPTX 604 kb) [file 12968_2019_562_MOESM4_ESM.pptx]
